# The application of gargle containing honeysuckle and semen oroxyli to reduce the pain and complications after uvulopalatopharyngoplasty

**DOI:** 10.3389/fphar.2022.974233

**Published:** 2022-10-17

**Authors:** Lihua Ren, Jun Li, Zhongyi Miao, Ru Yan, Qunzhen Li, Rong Zhang, Ting Rong, Xuena Dong

**Affiliations:** Department of Otorhinolaryngology Head and Neck Surgery, Hebei Provincial Hospital of Traditional Chinese Medicine, Hebei, China

**Keywords:** obstructive sleep apnea, uvulopalatopharyngoplasty, honeysuckle, semen oroxyli, pain, complications, VAS scale

## Abstract

**Purpose:** The main purpose of this pilot study was to detect the positive effects of our self-made gargle containing honeysuckle and semen oroxyli on post-operative pain and complications after Uvulopalatopharyngoplasty (UPPP).

**Methods:** Patients with obstructive sleep apnea (OSA) who had underwent elective UPPP during the period of April 2019 to January 2022 were randomly divided into treatment group and control group. The patients in the treatment group were instructed to gargle the solution containing honeysuckle and semen oroxyli (25 ml) four times a day for 2 weeks. The patients in the control group were instructed to gargle normal saline (25 ml) at the same schedule. The post-operative resting throat pain, swallowing throat pain and patient comfort level were evaluated at 12 h, 1 week and 2 weeks after UPPP. The post-operative complications were collected and analyzed.

**Results:** During the study period, 218 patients completed all the post-operative assessments. For resting throat pain, the treatment group had much lower VAS scores at 1 week and 2 weeks after UPPP. For swallowing throat pain, treatment group had much lower VAS scores than control group at 2 weeks after surgery. And patients in the treatment group felt more comfortable comparing to those in control group at 1 week and 2 weeks after UPPP. Although the treatment group had less numbers of patients with post-operative wound infection or bleeding, the difference between the 2 groups was not statistically significant.

**Conclusion:** The gargle containing honeysuckle and semen oroxyli could relieve both resting and swallowing throat pain and increase patient comfort after UPPP.

## Introduction

Obstructive sleep apnea (OSA) is a common disease characterized by repetitive upper airway collapse during sleep (Lee et al., 2021). Over 4% of men and 2% of women in the world are affected by this disease (Franklin and Lindberg, 2015). Untreated OSA is associated with a series of serious cardiovascular complications such as hypertension, coronary artery disease and heart failure, with increased mortality (Huang et al., 2021). Various neurodegenerative disorders have also been suspected to be associated with OSA (Lee et al., 2021).

Uvulopalatopharyngoplasty (UPPP) is the major treatment for OSA, which could relieve the obstruction of upper airway *via* shortening the uvula and soft palate or in company with tonsillectomy (Huang et al., 2021). The efficacy of UPPP to treat OSA has been demonstrated in two former randomized controlled trials (Browaldh et al., 2013; Sommer et al., 2016). However, post-operative pain and complications after UPPP may negatively impact the quality of life (QOL) and postoperative comfort of patients, leading to delayed recovery. Thus, finding a strategy to relieve post-operative pain and complications is important for perioperative management of UPPP.

Honeysuckle (*Lonicera japonica* Thunb) is a semi-evergreen vine shrub that grows in China, Japan and Korea (Ryu et al., 2010). It is well-known Chinese botanical drug, which has been long used as anti-bacterial, anti-viral, anti-oxidative and anti-inflammatory agents (Lin et al., 2019). The anti-inflammatory and analgesic activities of honeysuckle have been demonstrated in a variety of disease models (Ryu et al., 2010). It is a good candidate of phytomedicine to relieve pain and inflammatory. In China, honeysuckle is often used to treat throat pain. Semen Oroxyli is the seed of *Oroxylum indicum (L.) Vent*., which grows widely in the tropic regions in China (Yin et al., 2019). Modern pharmacological studies have demonstrated that semen oroxyli possesses antioxidant and anti-inflammatory activities (Yan et al., 2014). It is often used to treat respiratory disorders including coughs, chronic pharyngitis, and upper respiratory tract infections (Yan et al., 2014; Yin et al., 2019).

As mentioned above, both of honeysuckle and semen oroxyli have antioxidant and anti-inflammatory activities. Thus, the gargle containing honeysuckle and semen oroxyli has the potential to relieve pain and complications (such as wound infection and wound bleeding) after UPPP. The main purpose of this pilot study was to detect the positive effects of our self-made gargle containing honeysuckle and semen oroxyli on post-operative pain and complications after UPPP.

## Materials and methods

This study was registered at https://register.clinicaltrials.gov (registration number: ClinicalTrials.gov Identifier: NCT05535179). We used a Revised Cochrane risk-of-bias tool for randomized trials (RoB 2) to evaluate the methodological quality of the present study (See Supplementary Material).

### Preparation of gargle

Honeysuckle (*Lonicera japonica* Thunb), Semen oroxyli (*Oroxylum indicum* L), *Prunella vulgaris* (L), Wax gourd (*Benincasa hispida*) peel, Euchresta japonica (*Sophora tonkinensis* Gagnep.), *Rehmannia glutinosa* (*Rehmannia glutinosa* Libosch.) and Arnebiae Radix [*Arnebia euchroma* (Royle) Johnstn]. were obtained from Zhang Zhongjing Pharmacy (Henan, China). Honeysuckle (20 g), Semen oroxyli (15 g), Prunella vulgaris (30 g), Wax gourd (Benincasa hispida) peel (20 g), Euchresta japonica (6 g), *Rehmannia glutinosa* (10 g) and Arnebiae Radix (10 g) were ground into powder using a high-speed tissue crusher (AF-10A; Auari, Wenling, China). 15 g of mixed medicinal powder was totally dissolved in 500 ml of hot water.

### Patients

This was an open-label randomized controlled study which was approved by Institutional Review Board of our hospital. Patients with OSA, aged 18–60 years, who had undergone elective UPPP during the period of April 2019 to January 2022 were enrolled in this study. All of them signed the informed consents to publish the data.

In this study, OSA was diagnosed through polysomnography according to the international classification of sleep disorders approved by the America Academy of Sleep Medicine (Huang et al., 2021). The exclusion criteria were as followed: (Lee et al., 2021): patients with a history of systemic diseases such as severe cardiac and/or pulmonary disorders; (Franklin and Lindberg, 2015); patients who were unable to cooperate with evaluations; (Huang et al., 2021); patients with severe diabetes; (Sommer et al., 2016); patients who had underwent other surgeries in the last 6 months.

The patients were randomly divided by a computer-generated simple randomization schedule into treatment group and control group in a 1:1 ratio.

### Surgery

All patients in this study used Chengdu Meichuang low-temperature plasma surgery system PLA-600.

The operation was conducted through nasal intubation and general anesthesia. The disposable plasma surgical electrode tip: MC401 was selected.

Use a low temperature plasma knife to stop bleeding or cut. First, remove the tonsils on both sides, and cut the soft palate mucosa along both sides of the uvula in an inverted “U” shape.

The excessive fat tissue in the palatine velum space was ablated with plasma, and the palatopharyngeal arch and soft palate nasopharyngeal mucosa were preserved to avoid damaging muscle tissue (especially levator veli palatini and tensor veli palatini) as much as possible. The long and thick uvula tissue was partially ablated to fully stop bleeding.

Close the palatoglossal arch and palatopharyngeal arch mucosa on both sides with 4-0 absorbable suture, and clear the dead space. Avoid eating hard, overheated and irritating food after operation.

### Treatments

The patients in both groups were given first generation or second generation cephalosporins for 3–5 days after UPPP. Except for these drugs, patients in the treatment group were instructed to gargle the suspension solution containing honeysuckle and semen oroxyli (25 ml) for 5 min. The solution was given 4 times a day for 2 weeks. The patients in the control group were instructed to gargle normal saline (25 ml) at the same schedule. No NSAIDs were administered unless requested by the patient.

### Data collection

The post-operative resting throat pain and swallowing throat pain were evaluated at 0 weeks (12 h after surgery), 1 week and 2 weeks after UPPP by the patients themselves using a visual analog scale (VAS) based on a linear scale from 0 to 10, where 0 represented an absence of pain and 10 represented maximal pain. Then, the changes of scores from week 0 to week 2 between the 2 groups were compared. The VAS scores related to patient comfort level were also evaluated by patients themselves, with 0 representing very much worse and 10 very much comfort. Furthermore, the researchers who were blinded to the therapeutic regimens evaluated the improvement in postoperative pain of patients within the 2-weeks postoperative period based on the clinical global impression of improvement (CGI-I score) questionnaire. In this questionnaire, the researchers used a seven-point scale from 1 (very much improved) to 7 (very much worse) to rate the improvement of body pain of the patients (Huang et al., 2009).

The post-operative complications, such as wound infection and wound bleeding, were collected and analyzed. The baseline characteristics of the patients in 2 groups including age, gender, body mass index (BMI), OSA severity, American Society of Anesthesiologist (ASA) classification and pre-operative complications were also collected. The venous blood was collected at 12 h and 1 week after operation to detect the levels of hs-CRP, hemoglobin, neutrophil% and white blood cell count (WBC).

### Statistical analysis

The data in this study were analyzed by the SPSS software (version 22.0). Normal distribution quantitative data were described as mean ± SD. The difference between groups were compared by the Student’s t test. Non-normal distribution quantitative data were described as median with range and compared with Mann-Whitney U test. Categorical data were described as numbers and percentages and compared using the Chi-square test or Fisher’s exact test. *p* < 0.05 was considered as statistical significance.

## Results

**FIGURE 1:**
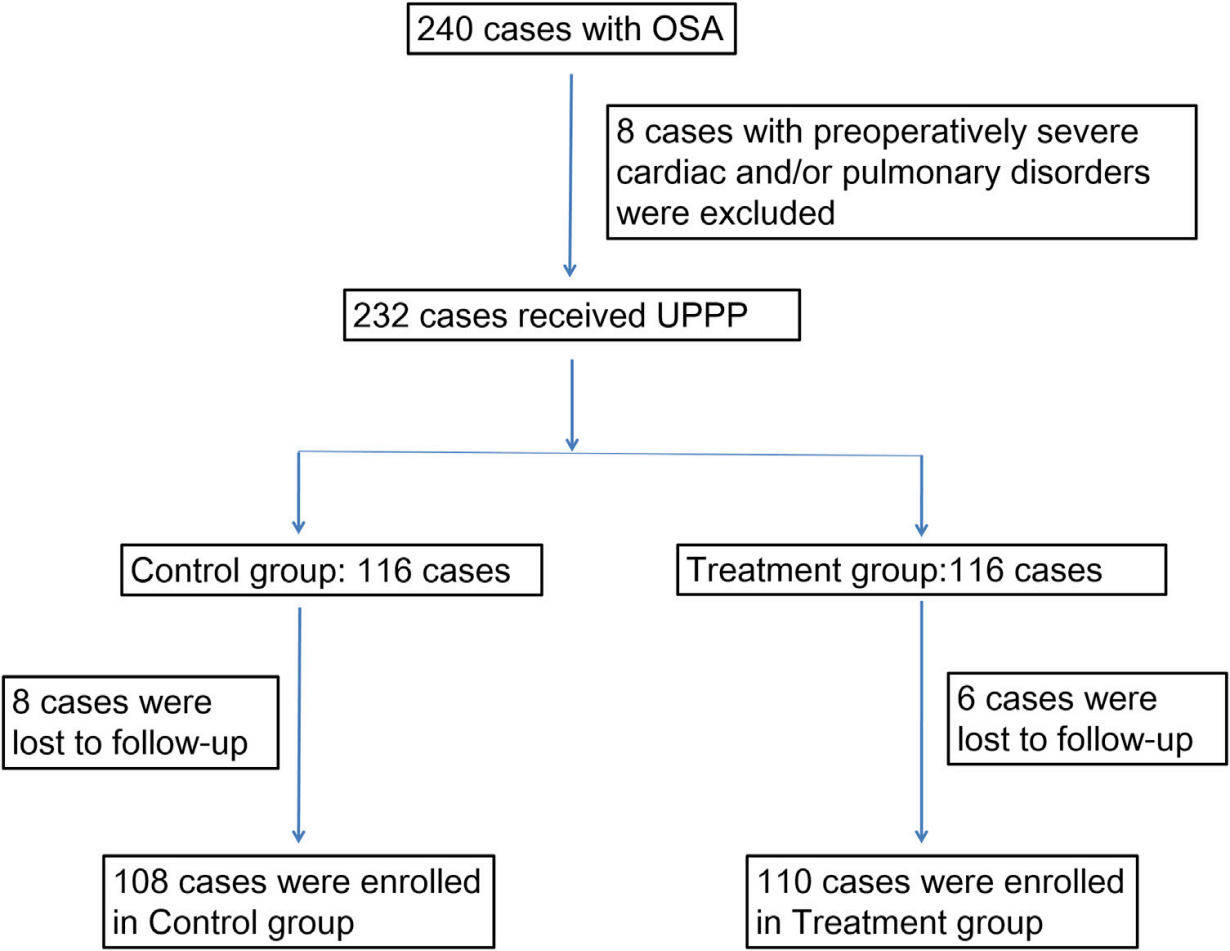
Study profile.

During the study period, 240 patients with OSA were screened in this study (Figure 1). 8 of them were excluded for preoperatively severe cardiac and/or pulmonary disorders. 232 were randomly divided into treatment group (*n* = 116) and control group (*n* = 116). However, 6 patients in the treatment group and 8 patients in the control group were lost to follow-up. Finally, 218 patients completed all the post-operative assessments (*n* = 110 and 108 for treatment and control groups, respectively), the data of whom were included into the statistical analysis. The characteristics of the 2 groups are shown in Table 1.

**TABLE 1:** The characteristics of the patients enrolled in this study.

Characteristic	Treatment group (n = 110)	Control group (n = 108)	*p* Value
Gender, n (%)			0.306
Male	97 (88.18)	90 (83.33)	
Female	13 (11.82)	18 (16.67)	
Age (years, mean ± SD)	43.24 ± 10.65	41.56 ± 11.47	0.264
BMI (kg/m^2^, mean ± SD)	26.32 ± 4.30	26.74 ± 3.78	0.445
OSA severity, n (%)			0.448
Mild	15 (13.64)	9 (8.33)	
Moderate	33 (30.00)	36 (33.33)	
Severe	62 (56.36)	63 (58.33)	
ASA classification n (%)			0.595
Class I	51 (46.36)	50 (46.30)	
Class II	54 (49.09)	56 (51.85)	
Class III	5 (4.55)	2 (1.85)	
NYHA classification, n (%)			0.803
Class I	95 (86.36)	92 (85.19)	
Class II	15 (13.64)	16 (14.81)	
Pre-operative complications, n (%)			
Hypertension	42 (38.18)	36 (33.33)	0.455
Diabetes	14 (12.72)	11 (10.18)	0.556
Surgical duration (min, mean ± SD)	61.04 ± 17.71	61.18 ± 17.81	0.954
0.954Intraoperative in-put (ml, mean ± SD)	749.49 ± 10.55	748.52 ± 9.51	0.476
Intraoperative bleeding (ml, mean ± SD)	15.55 ± 4.53	15.58 ± 4.48	0.951
Patients used NSAIDs (n, %)	43 (39.29)	45 (41.67)	0.062
Post-anesthesia care unit stay (min, mean ± SD)	29.71 ± 4.62	28.70 ± 5.95	0.164
Total length of stay (day, median (IQR))	8 (Yan et al., 2014; Lin et al., 2019; Yin et al., 2019)	8 (Yan et al., 2014; Lin et al., 2019; Yin et al., 2019)	0.740
Postoperative length of stay (day, median (IQR))	5 (Ryu et al., 2010; Browaldh et al., 2013; Sommer et al., 2016)	5 (Ryu et al., 2010; Browaldh et al., 2013; Sommer et al., 2016; Lin et al., 2019)	0.816

BMI, body mass index; OSA, obstructive sleep apnea; ASA, american society of anesthesiologists; NYHA, new york heart association; IQR, interquartile range.

For resting throat pain at 1 week and 2 weeks after UPPP, the VAS scores of the treatment group were much lower than those of the control group (*p* = 0.033 and *p* = 0.031, respectively, Table 2). However, the VAS scores for resting throat pain measured at 12 h after UPPP were not significantly different between the 2 groups (*p* = 0.465). For swallowing throat pain, treatment group had much lower VAS scores than control group at 2 weeks after surgery (*p* < 0.001). But the 2 groups had similar VAS scores for swallowing throat pain at 12 h and 1 week after surgery (*p* = 0.414 and 0.732, respectively, Table 2). Furthermore, patients in the treatment group felt more comfortable comparing to those in control group at 1 week and 2 weeks after UPPP (Table 2).

**TABLE 2:** The VAS score of postoperative pain and patient comfort.

	Treatment group (n = 110)	Control group (n = 108)	*p* Value
Resting throat pain, mean ± SD			
12 h after surgery	3.19 ± 1.16	2.93 ± 1.13	0.454
1 week after surgery	2.36 ± 1.03	2.80 ± 1.15	0.038
2 weeks after surgery	1.31 ± 0.99	1.74 ± 1.14	0.037
Swallowing throat pain, mean ± SD			
12 h after surgery	4.64 ± 1.30	4.85 ± 1.05	0.344
1 week after surgery	4.15 ± 1.04	4.22 ± 1.00	0.696
2 weeks after surgery	2.15 ± 1.11	2.98 ± 1.25	<0.001
Patient comfort, mean ± SD			
12 h after surgery	5.09 ± 1.16	4.93 ± 1.13	0.304
1 week after surgery	7.13 ± 0.75	6.22 ± 0.72	<0.001
2 weeks after surgery	7.60 ± 0.76	7.19 ± 0.73	0.004

**FIGURE 2:**
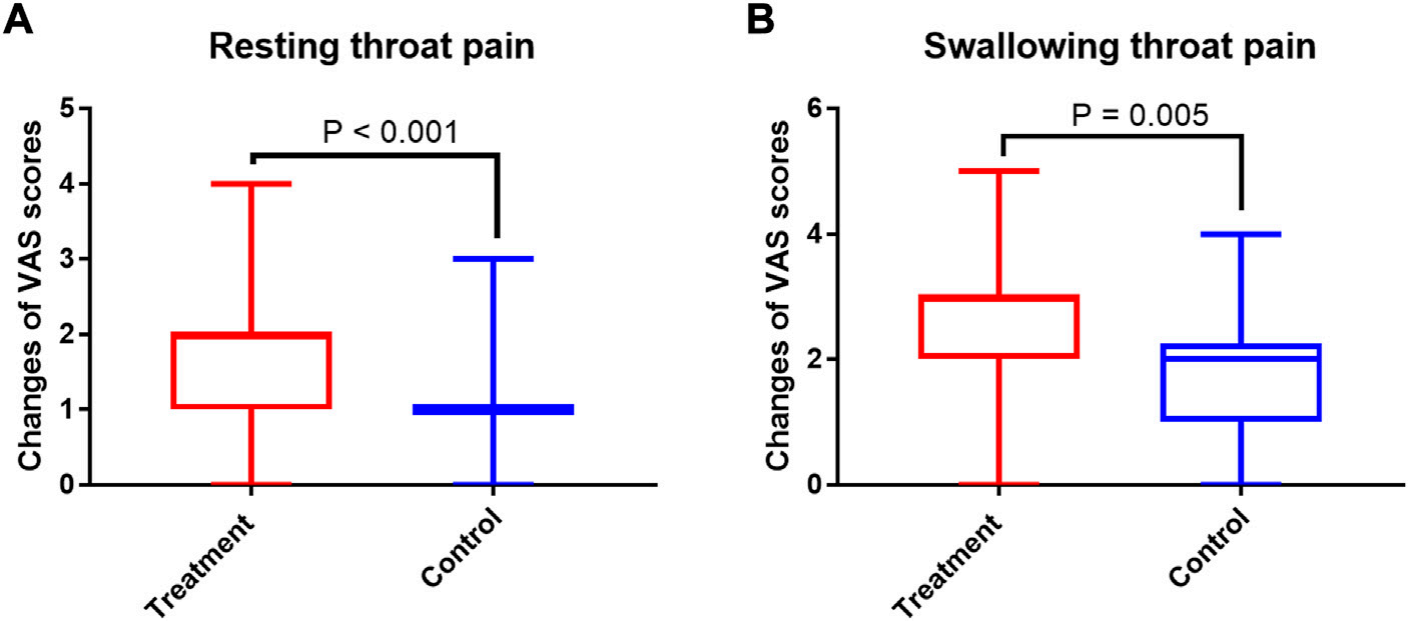
The comparisons of changes of VAS scores for resting throat pain **(A)** and swallowing throat pain **(B)**. VAS, visual analogue scales.

**FIGURE 3:**
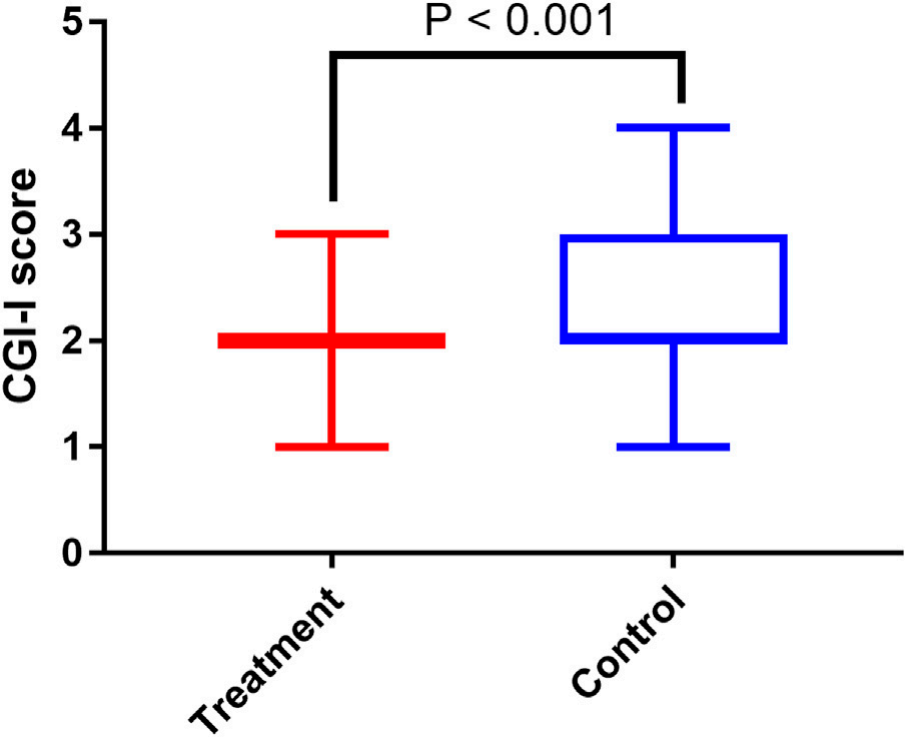
The comparison of CGI-I scores in treatment and control groups. CGI-I, clinical global impression of improvement.

As shown in Figure 2, the changes of VAS scores for both resting and swallowing throat pain from week 0 to week 2 were significantly higher in treatment group than in control group (*p* < 0.001 and *p* = 0.005, respectively). In addition, the treatment group performed a lower GGI-I score than control group (*p* < 0.001, Figure 3), indicating patients in treatment group had a higher improvement of body pain.

No deaths occurred within the 2-weeks postoperative period. The total complication rates between the 2 groups was not significantly different (*p* = 0.071). Although the treatment group had less numbers of patients with post-operative wound infection or wound bleeding, the difference between the 2 groups was not statistically significant (Table 3). The sub-analysis of complications is presented in Table 3.

**TABLE 3:** The comparison of complications between the 2 groups.

	Treatment group (n = 110)	Control group (n = 108)	*p* Value
Total complications, n (%)	18 (16.36)	29 (26.85)	0.071
Wound infection, n (%)	2 (1.82)	5 (4.63)	0.278
Wound bleeding, n (%)	6 (5.45)	12 (11.11)	0.129
Hypoxia, n (%)	0 (0)	1 (0.93)	0.495
Dizziness, n (%)	7 (6.36)	6 (5.56)	0.514
Nausea and vomiting, n (%)	3 (2.72)	5 (4.63)	0.497

The levels of hs-CRP, hemoglobin, neutrophil% and WBC between the 2 groups at 12 h after surgery were not significantly different. 1 week after operation, the levels of hs-CRP, Hemoglobin, and WBC in the treatment group were significantly lower than those in the control group (*p* < 0.05, Table 4).

**TABLE 4:** Comparison of inflammatory factor levels between two groups.

	Treatment group (n = 110)	Control group (n = 108)	*p* Value
Hs-CRP (ng/ml)			
12 h after surgery	2.35 ± 0.46	2.24 ± 0.43	0.067
1 week after surgery	1.41 ± 0.45	1.93 ± 0.50	<0.001
Hemoglobin (g/L)			
12 h after surgery	120.76 ± 20.03	121.05 ± 19.06	0.911
1 week after surgery	105.23 ± 14.96	116.16 ± 20.71	<0.001
Neutrophil %			
12 h after surgery	75.66 ± 9.96	77.67 ± 9.12	0.123
1 week after surgery	69.64 ± 10.08	72.28 ± 10.64	0.062
WBC (×10^9^/L)			
12 h after surgery	9.30 ± 2.20	8.89 ± 2.06	0.160
1 week after surgery	6.86 ± 1.39	8.78 ± 1.61	<0.001

## Discussion

Currently, UPPP is still the most commonly performed surgery for OSA (Lee et al., 2021). However, the pain and complications after UPPP negatively impact the QOL of patients. Previous studies have showed that poorly controlled post-operative pain will reduce patient satisfaction and cause adverse consequences (Gan, 2017). Thus, finding a strategy to relieve post-operative pain and complications is necessary. In the present study, we found that the gargle containing honeysuckle and semen oroxyli could relieve both resting and swallowing throat pain after UPPP. But this gargle might not reduce the occurrence of post-operative complications.

Post-operative pain is one of the important symptoms after UPPP (Zodpe et al., 2006). The pain can often occur at throat and ears and can be continued until mucosa is complete recovery (Zodpe et al., 2006). The current research on pain and analgesia after OSA mostly focuses on the early 24–48 h after UPPP. However, it was clinically observed that pharyngeal pain persisted within 1 week after surgery, which seriously affected the quality of life of patients. There are various ways to relieve pain after UPPP, including local injection and systemic administration. Commonly used local analgesia is the application of local anesthetics, such as ropivacaine, before mucosal incision. But limited by the half-life of the drug, its effective time is usually 4–6 h (Ghezal et al., 2020). In addition, NSAIDs commonly have gastrointestinal reactions, especially the occurrence of peptic ulcers (García-Rayado et al., 2018). Natural medicines have the advantages of wide range of materials, safety, and less toxic and side effects, and have great advantages and potential in the prevention and treatment of oral diseases. Due to the excellent anti-inflammatory effect and low toxicity, we included honeysuckle and semen oroxyli in our self-made gargle with the aim to relive the pain and complications after UPPP. Actually, we did find that using the gargle containing honeysuckle and semen oroxyli could relieve both resting and swallowing throat pain after UPPP. Our results showed that treatment group had much lower VAS scores for resting throat pain at 1 week and 2 weeks after UPPP comparing to the control. For swallowing throat pain, treatment group had much lower VAS scores than control group at 2 weeks after surgery. Furthermore, the changes of VAS scores for both resting and swallowing throat pain from week 0 to week 2 were significantly higher in treatment group than in control group. Except for the VAS scores measured by the patients themselves, the GGI-I scores evaluated by the researchers also favored to treatment group, indicating patients in treatment group had a higher improvement of body pain. Because of the effect of our gargle to relieve pain, patients in the treatment group felt more comfortable after UPPP.

We found that the levels of hs-CRP, hemoglobin and WBC in the treatment group at 1 week after surgery were significantly lower than those in the control group, suggesting the anti-inflammatory effect of the mouthwash containing honeysuckle and semen oroxyli on UPPP. Inflammation plays an important role in post-operative pain occurrence. The inflammatory mediators produced during inflammation, including cyclooxygenase-2 (COX-2) and interleukin-6 (IL-6), can evoke pain *via* direct activation and sensitization of nociceptors (Matsuda et al., 2019). Honeysuckle is widely used in China as an anti-inflammatory and anti-oxidative agent. Previous studies have revealed that honeysuckle could inhibit various inflammatory factors exerting strong effect against bacteria induced inflammatory mediators (Shang et al., 2011; Kao et al., 2015). Lin et al. (2021) found that honeysuckle water extract could reduce the expression of proinflammatory mediators through NF-κB pathway. Ryu and colleague found that the active ingredients from honeysuckle exerted anti-inflammatory and analgesic effects by inhibition of COX-2 induction and activity of iNOS (Ryu et al., 2010). Furthermore, through the inflammation and pain models in mice, Li et al. (2021) found that the extractions from honeysuckle possessed excellent pharmacological activities such as inhibiting inflammation and oxidation, and relieving pain. The other constitute of our gargle, semen oroxyli, also possesses antioxidant and anti-inflammatory activities. The study conducted by Lalrinzuali et al. (2016) indicated that semen oroxyli possesses both anti-inflammatory and analgesic activities and it could be used as an anti-inflammatory agent. Sithisarn et al. (2016) found that the extracts from semen oroxyli had an *in vitro* antioxidant effect, with anti-bacterial potential. The anti-inflammatory and analgesic activities of honeysuckle and semen oroxyli may contribute to these results.

After UPPP, oral flora may increase. It can cause infection and inflammation in the throat, resulting in more pain in advance of infection (Zodpe et al., 2006). And the infection could also result in the secondary postoperative bleeding (Zodpe et al., 2006). Thus, the wound infection and wound bleeding are the post-UPPP complications we should concern. Previous studies showed that both of honeysuckle and semen oroxyli possessed anti-bacteria and anti-inflammatory abilities (Yan et al., 2014; Lin et al., 2019). Thus, in theory, our self-made gargle could prevent the occurrence of post-UPPP complications. However, in the present study, we observed that the gargle did not reduce the numbers of patients with wound infection or bleeding after UPPP. It has been reported that occurrence rate of post-UPPP wound infection or bleeding is very low (Huang et al., 2021). Thus, to determine the preventive effect of the gargle containing honeysuckle and semen oroxyli on post-UPPP wound infection or bleeding, we need larger sample size.

The present study has several limitations. First, this study was carried out in a single center with small sample size. A multi-center with a larger sample is needed to confirm the conclusions of this study. Second, the assessment of post-UPPP pain based on VAS was completely subjective. More objective methods should be considered in the further studies. Third, in this study, we used the theory behind the VAS to evaluate patient comfort. It is not a widely accepted method. Thus, more precise evaluation of patient comfort should be applied in future studies. Forth, clinical application needs to consider the individual differences of patients and the safety and effectiveness of drugs. Therefore, further research is still needed for the promotion of this homemade mouthwash. Finally, we did not detect the mechanisms why the gargle containing honeysuckle and semen oroxyli could relieve post-UPPP pain. The mechanisms should be investigated in our future studies.

## Conclusion

In conclusion, our results found the gargle containing honeysuckle and semen oroxyli could relieve both resting and swallowing throat pain and increase patient comfort after UPPP in patients with OSA.

## Data Availability

The original contributions presented in the study are included in the article/supplementary material, further inquiries can be directed to the corresponding author.
